# Development of PMAxx-droplet digital PCR method for the absolute quantification of viable *Shigella flexneri* and *Shigella sonnei* strains in water

**DOI:** 10.3389/fmicb.2026.1769049

**Published:** 2026-03-06

**Authors:** Xuran Zhang, Chunmei Liu, Mimi Kong, Ziqiang He, Zhijie Cao, Dong Jin

**Affiliations:** 1 National Institute for Communicable Disease Control and Prevention, Chinese Center for Disease Control and Prevention, Beijing, China; 2 Linfen Center for Disease Control and Prevention, Linfen, Shanxi, China; 3 Qiaoxi District Center for Disease Control and Prevention, Shijiazhuang, Hebei, China; 4 Hebei Key Laboratory of Intractable Pathogens, Shijiazhuang Center for Disease Control and Prevention, Shijiazhuang, Hebei, China

**Keywords:** droplet digital PCR, PMAxx, *Shigella flexneri*, *Shigella sonnei*, water

## Abstract

**Introduction:**

*Shigella spp*. are waterborne pathogens responsible for global diarrheal disease outbreaks via contaminated groundwater, drinking water, and recreational water systems. Their extremely low infectious dose necessitates the development of highly sensitive detection methods capable of distinguishing viable pathogens.

**Methods:**

In this study, we developed and optimized a viability-discriminative droplet digital PCR (ddPCR) assay incorporating propidium monoazide (PMAxx) treatment and duplex amplification targeting both chromosomal and virulence plasmid genes. Key reaction parameters—including annealing temperature, primer and probe concentrations, and PMAxx treatment conditions—were systematically optimized. Singleplex and duplex assays were compared to verify amplification consistency. Additionally, three DNA concentration methods (direct centrifugation, PEG precipitation, and a commercial kit) were evaluated for their suitability in field applications using fecal-spiked water samples.

**Results:**

The optimized PMAxx-ddPCR assay enabled simultaneous detection of viable *S. flexneri* and *S. sonnei* with excellent specificity. Duplex amplification showed amplification efficiencies comparable to those of singleplex assays across all targets. The method achieved a detection limit of ≤10 CFU/reaction in fecal-spiked water, and PMAxx treatment effectively suppressed signals from dead cells. Comparative evaluation of concentration methods identified effective protocols suitable for field deployment.

**Conclusion:**

This PMAxx-ddPCR approach enables the simultaneous quantification of viable, virulent *Shigella* in water samples, offering a robust tool for advancing water safety monitoring and public health protection.

## Introduction

Waterborne transmission serves as a critical dissemination pathway for enteric bacterial pathogens including *Salmonella*, *Shigella*, and *Escherichia coli* (Kunz et al., 2024; World Health Organization, 2025). The pathogen demonstrates remarkable environmental persistence in aquatic systems, particularly in inadequately chlorinated community water sources (McClung et al., 2020). Transmission dynamics exhibit seasonal variation, with outbreaks peaking during rainy seasons characterized by elevated temperatures and increased rainfall (Wang et al., 2021). These conditions facilitate surface runoff contamination and create favorable conditions for bacterial proliferation (Lim and Foo, 2021). While chlorination remains the primary disinfection method in water treatment facilities, particularly in developing countries, it does not ensure complete pathogen elimination (LeChevallier et al., 2024).

*Shigella* are major waterborne pathogens (World Health Organization, 2025). Waterborne transmission represents a critical pathway for *Shigella* dissemination, with contamination occurring through sewage-polluted groundwater, drinking water systems, and recreational water bodies (Fleming et al., 2000; Kunz et al., 2024; World Health Organization, 2025). Epidemiological surveillance in the United States (1997–2022) documented 60 splash pad-associated outbreaks, including five confirmed *Shigella* infection cases among 52 laboratory-verified incidents (Lawinger et al., 2024), highlighting the public health significance of viable pathogen detection in aquatic matrices. The ability to quantify live *Shigella*—particularly plasmid-bearing strains—carries profound implications for evaluating water treatment efficacy, tracing outbreak sources, and validating sanitation interventions. Current microbial risk management requires integrated water safety plans that address fecal contamination along entire supply chains, enforce recreational water standards, and target *Shigella* persistence hotspots through advanced monitoring technologies.

*Shigella*, a genus of facultative anaerobic Gram-negative bacteria, represents a major etiological agent of bacterial dysentery, contributing significantly to global morbidity and mortality, particularly in children under 5 years of age (Kotloff et al., 2018). The fecal-oral transmission of shigellosis occurs primarily through contaminated food and water, with an exceptionally low infectious dose of 10–100 bacterial cells. Globally, *Shigella* infections rank as the second leading cause of diarrheal mortality, accounting for approximately 210,000 deaths annually, including 63,700 deaths in children under five (Khalil et al., 2018). The genus *Shigella* comprises four species distinguished by O-antigen structure: *Shigella dysenteriae*, *Shigella flexneri*, *Shigella sonnei*, and *Shigella boydii*. Epidemiological surveillance reveals that *S. flexneri* and *S. sonnei* constitute the primary contributors to the global burden of shigellosis. While *S. flexneri* predominates in low- and middle-income countries, *S. sonnei* represents the most prevalent serogroup in high-income nations (Kotloff et al., 2018). Notably, resistant clones have emerged across diverse geographical and socioeconomic contexts. A concerning example is the *S. sonnei* clone demonstrating resistance to ceftriaxone and azithromycin caused waterborne outbreaks in China between 2015 and 2020, highlighting its evolutionary advantage and transmission potential (Qiu et al., 2022). The development of sensitive detection methods is crucial given the potentially low concentrations of *Shigella* in water systems. Furthermore, the ability to determine both bacterial viability and the presence of virulent plasmids is essential for accurate risk assessment of contaminated water sources.

Droplet digital PCR (ddPCR) represents a significant advancement over conventional PCR methods, offering enhanced sensitivity, specificity, and reproducibility. Unlike quantitative PCR (qPCR), which relies on standard curves for relative quantification, ddPCR partitions the sample into thousands of nanoliter-sized droplets and employs Poisson distribution statistics to achieve absolute quantification without the need for external calibration (Kojabad et al., 2021). This capability for direct, absolute nucleic acid counting establishes ddPCR as a reference method for the quantification and value assignment of nucleic acids in analytical science (Li et al., 2025). In the field of pathogen detection, its exceptional sensitivity and high tolerance to PCR inhibitors make it particularly valuable for analyzing samples with low pathogen loads or high levels of background interference (Ottesen et al., 2006; Cao et al., 2025). However, similar to other molecular detection methods, ddPCR cannot inherently distinguish between viable and non-viable bacterial cells at the DNA level. This limitation can be addressed through propidium monoazide (PMA) staining, a widely adopted technique for selective detection of viable pathogens. PMA selectively binds to DNA from membrane-compromised cells, inhibiting their amplification while maintaining the detectability of viable cells (Kallastu et al., 2023). The non-toxic nature of PMA and the variant PMAxx appropriate concentrations further enhances its utility for viability assessment (Mancabelli et al., 2021).

This study presents the development and optimization of a novel PMAxx-ddPCR detection method through systematic evaluation of key parameters, including annealing temperature, primer concentration, probe concentration, PMAxx concentration, and PMAxx light exposure duration. The developed protocol enables simultaneous quantification of viable *Shigella* in water samples through parallel detection of large virulence plasmids and chromosomal genes, providing a robust tool for water safety assessment and microbial risk analysis.

## Materials and methods

### Primers and probes

Three sets of primers and probes were designed using Primer Express 3.0 software (Applied Biosystems, CA, USA) and were synthesized by Beijing Tianyi Huiyuan Biotechnology (Beijing, China) to target specific genes unique to *S. flexneri* and *S. sonnei* strains used in this study. These primers and probes were developed based on three single-copy genes: *set*1A, *ss_methylase*, and *ipaBCD* (Faruque et al., 2002; Wu et al., 2019). Two duplex digital PCR (ddPCR) reaction systems were established to enable simultaneous quantification of chromosomal and large virulence plasmid copy numbers in *S. flexneri* and *S. sonnei* strains. Specifically, the *set1* gene is located on the chromosome of *S. flexneri*, the *ss_methylase* gene is present on the chromosome of *S. sonnei*, and the *ipaBCD* gene is located on the large virulence plasmid shared by both *S. flexneri* and *S. sonnei* (Table 1).

**Table 1:** The primers and probes used in this study.

Target	Genes	Sequence and modification (5′-3′)	Amplicon size (bp)
*Shigella flexneri*	*set*1A	F:CCGGCCAGGATCCTATCTTT	60
		R:GGGAGTTTGTTGGCATGAACA	
		P:FAM-ACTGTCTGAACTGCTTTG-BHQ1	
*Shigella sonnei*	*ss_methylase*	F:CGGGCAGTCAAGGTTGATG	64
		R:CAATATGCAGCTACGATGCTGAA	
		P:FAM-TGCCCGCTCATACTG-BHQ1	
*Shigella*	*ipaBCD*	F:GCAAAAGCGACAGCATAAAGG	72
		R:GGGACTCGCAGCTATTTATCAGA	
		P:HEX-TGCTGCTTGTTGGAAC-BHQ1	

To confirm the specificity of the primers and probes, we utilized the BLASTn algorithm and Primer-BLAST tools available through NCBI BLAST.^1^ The common PCR products of *set1*, *ss_methylase*, and *ipaBCD* genes which were extracted from the gel using QIAquick Gel Extraction Kit (Qiagen, Hilden, Germany) were cloned into the pMD18-T vector (Takara, Dalian, China). Recombinant plasmids containing three target genes were purified using the Plasmid Mini Kit (Omega, GA, USA) and quantified via Qubit 4 Fluorometer with dsDNA HS Assay Kit (Thermo Fisher Scientific, MA, USA). Sanger sequencing verification was performed by Beijing Tianyi Huiyuan Biotechnology using universal M13 primers. The copy number concentration of the recombinant plasmid standard was derived from its mass concentration using the standard conversion formula: copies/μL = [DNA concentration (ng/μL) × 10^−9^ × Avogadro’s number] / [660 × total plasmid size (bp)], with plasmids being widely used as quantitative standards in qPCR assays (Shin et al., 2024).

### Bacterial strains and preparation of DNA templates

The *S. flexneri* strain 301 and *S. sonnei* strain CMCC51081 were used as the primary strains for method evaluation in this study. Additionally, a panel of 82 reference and clinical strains including 53 *Shigella* strains was included to assess the specificity of the ddPCR assay (Supplemental Table 1). To confirm the presence of large virulence plasmids in the *Shigella* strains, Congo Red agar plates were employed. Only red colonies were selected for further analysis (Smith et al., 1990).

### Real-time PCR assay

Fluorescence quantitative PCR was performed on a Rotor-Gene Q instrument (Qiagen, Hilden, Germany) with Premix Ex Taq (Probe qPCR) (Takara, Dalian, China). Each 20 μL reaction contained 10 μL of 2 × Premix Ex Taq, 0.4 μL each of forward and reverse primers (10 μM), 0.2 μL of probe (10 μM), and 2 μL of DNA template. Nuclease-free water was added to bring the total volume to 20 μL. Thermal cycling was conducted under the following conditions: initial denaturation at 95 °C for 30 s, followed by 40 cycles of denaturation at 95 °C for 5 s and combined annealing/extension at 60 °C for 30 s. The threshold for qPCR was set to the value which above the background noise and within the exponential phase of amplification curves, ensuring accurate determination of Cycle Threshold (Ct) values. Tenfold serial dilutions of the recombinant plasmid standard were prepared to generate a concentration range of 1.0 × 10^0^ to 1.0 × 10^8^ copies/μL, encompassing nine discrete concentration levels. Each dilution was analyzed in triplicate. Ct values were plotted against log concentrations, and the coefficient of determination (*R*^2^) was calculated using linear regression analysis to assess linearity.

### ddPCR assay

The ddPCR experiments were conducted using a QX200™ ddPCR system (Bio-Rad, CA, USA). Each ddPCR reaction consisted of a 20 μL reaction mixture, which included 10 μL of 2 × ddPCR™ SuperMix for Probes (Bio-Rad), 500 nM of probes, 500 nM of forward and reverse primers were used for single PCR reaction, 2 μL of template DNA, and nuclease-free water to reach a final volume of 20 μL. Once the reaction mixture was prepared, droplets were generated using a DG8 cartridge and the droplet generator (Bio-Rad). These droplets were then transferred to a 96-well plate and sealed using a PX1 PCR plate sealer (Bio-Rad). The thermal cycling protocol involved an initial denaturation step at 95 °C for 10 min, followed by 45 cycles of denaturation at 94 °C for 30 s and annealing/extension at different temperatures for 1 min. A final extension was performed at 98 °C for 10 min. The amplification products were then held at 4 °C for 30 min. The temperature ramp speed was set at 2 °C/s. Following PCR amplification, plates were read on the QX200 Droplet Reader. Data was analyzed using QuantaSoft software (QX Manager Software 2.2), where thresholds between positive and negative droplet populations were set manually based on the fluorescence amplitude gap of negative controls. Only wells with >10,000 accepted droplets were included in the analysis. The limit of blank (LoB) for each target gene was determined by testing 35 blank samples (ddH₂O), as detailed in Supplemental Table 2. The distribution of the results was confirmed to be non-normal by the Shapiro–Wilk test. The LoB was then calculated by ranking the results in ascending order, identifying the 95th percentile rank, and performing linear interpolation according to the non-parametric method recommended in the CLSI EP17 guideline. A ddPCR readout exceeding the LoB value was considered positive detection.

### Optimization and comparison of Singleplex and duplex ddPCR assays

Systematic optimization of singleplex ddPCR assays was performed across three critical parameters: annealing temperature, primer concentrations, and probe concentrations (Liu et al., 2024). To determine the optimal annealing temperature for the primer-probe set targeting the *ipaBCD* gene, a gradient of 10 temperatures ranging from 51.0 °C to 59.7 °C (specifically 51.0 °C, 51.5 °C, 52.3 °C, 53.2 °C, 54.3 °C, 55.4 °C, 56.6 °C, 57.7 °C, 58.8 °C, and 59.7 °C) was tested, using fixed concentrations of primers and probes. Primer concentrations were varied between 300 nM and 1,100 nM, and 12 probe concentrations were adjusted between 100 nM and 800 nM to optimize signal detection and specificity for *ipaBCD* gene. The genomic DNA extracted from *S. flexneri* strain 301 quantified at 49.5 pg./μL was used as the template. Duplex ddPCR assays were developed in a two-step process. First, the amplification efficiencies of all primer-probe sets were verified to be consistent using serial dilutions of plasmid standards in qPCR. Subsequently, the empirically optimized singleplex assays for *ipaBCD*/*set1A* and *ipaBCD*/*ss_methylase*, with equal primer and probe concentrations, were directly combined to establish the final duplex reaction systems.

### Specificity and sensitivity of ddPCR assay

To evaluate the specificity of the duplex ddPCR assay, reactions were conducted using DNA templates from 82 bacterial pathogen strains listed in the Supplemental Table 1. For sensitivity testing, the copy numbers of purified recombinant plasmids containing three target genes were calculated according as described previously (Feng et al., 2023). Nine decadal dilutions (10^8^–10^0^ copies/μL) were prepared in triplicate to determine the sensitivity of qPCR and ddPCR methods. Technical replicates were pooled prior to analysis to minimize volumetric errors.

### Optimization of PMAxx concentration and light exposure time

A systematic optimization for PMAxx (Biotium, CA, USA) treatment conditions was established using qPCR-based viability assessment modified from Hu et al. (2022). *S. flexneri* strain 301 was incubated in BHI broth overnight at 37 °C. The 50 μL strain solutions were then transferred to 5 mL of BHI broth and incubated until they reached an optical density (OD) ≈ 0.60 at 600 nm with continuous agitation. The strains were quantified by plate counting method. The *S. flexneri* strain 301 suspension (3.20 × 10^8^ CFU/mL) was first diluted to a working concentration of 3.20 × 10^6^ CFU/mL for the optimization of PMAxx treatment conditions. An aliquot of this diluted suspension was then heat-inactivated (80 °C, 20 min) to prepare the non-viable cell controls used in the optimization experiments. Eight PMAxx concentrations (0, 10, 20, 40, 60, 80, 100, and 120 μM) were evaluated to determine the optimal threshold that effectively suppressed DNA amplification from non-viable cells while maintaining detection sensitivity for viable cells. PMAxx solutions were incubated with bacterial suspensions in dark conditions (25 °C, 10 min) followed by photoactivation using the PMA-Lite™ LED Photolysis Device (Biotium, USA) or the 200 W blue LED lighting array with an output wavelength of 465 nm. The DNA of the strains was extracted using the QIAamp DNA Blood Mini Kit (Qiagen, NRW, Germany). A time-course experiment (0–30 min, 5 min increments) was conducted to determine the minimal exposure duration required for effective DNA cross-linking in non-viable cells. The Student’s *t*-tests and One-way ANOVA with Tukey’s *post hoc* testing were used to compare viable/non-viable cell groups and evaluated light exposure durations with statistical significance defined at *p* < 0.05. Biological replicates (*n* = 3) were analyzed as independent observations.

### Comparison and optimization of DNA extraction methods from simulative fecal contaminated water samples

The workflow for processing simulated fecal-contaminated water samples is shown in Supplemental Figure 1. A 10% (w/v) fecal suspension was prepared by homogenizing 1 g of healthy human donor stool in 9 mL sterile phosphate-buffered saline. The suspension was centrifuged at 2800 × *g* for 2 min to remove the sediment particulate matter. To prepare the fecal simulation, 500 μL of the fecal suspension was added to 50 mL of *Shigella*-free landscape water samples through pre-screening using qPCR method (Vu et al., 2004), creating a simulated fecal-contaminated water sample.

A standardized 50 mL aliquot of water was processed through three parallel concentration methods for comparative evaluation: direct centrifugation, polyethylene glycol (PEG) precipitation, and commercial kit-based filtration (Kit method). The 200 μL of bacterial solutions prepared by serial dilution and quantified by plate counting with different concentrations were mixed with simulated water samples. For the direct centrifugation method, samples were concentrated at 12000 × *g* for 5 min at 4 °C. The supernatant was carefully decanted, and the pellets were resuspended in 200 μL sterile ddH₂O, achieving a 1:100 concentration factor. For the PEG precipitation method, modified from Sapula et al. (2021), each 50 mL sample received 5.0 ± 0.1 g of polyethylene glycol 8,000 (PEG 8000, Sangon Biotech, Shanghai, China) and 1.13 ± 0.01 g of NaCl. Following 15 min of dissolution with vortex mixing samples were centrifuged at 10000 × *g* for 60 min at 4 °C with the brake disengaged. The resulting pellet was reconstituted in 200 μL of ddH₂O. For all concentrated samples, nucleic acids were purified using the QIAamp DNA Mini Kit (Qiagen) according to the manufacturer’s protocol. For the DNeasy PowerWater Kit (Qiagen), samples were vacuum-filtered through 0.45 μm polyethersulfone membranes using a Sterifil aseptic filtration system (Millipore, MA, USA). Membranes containing microbial biomass were transferred to PowerBead tubes containing 800 μL PW1 buffer and homogenized using the vortex for 10 min. The PowerWater Kit protocol was followed for comparative analysis, featuring sequential washes with PW2/PW3 buffers and inhibitor removal through silica-membrane chromatography. Biological replicates (*n* = 3) were treated as independent observational units. Amplification performance between direct centrifugation and kit-based methods was compared using Student’s *t*-tests, with statistical significance defined at *p* < 0.05.

### The sensitivity and application of PMAxx-ddPCR assay in simulative fecal contaminated water samples

*Shigella* concentrations were prepared by serial dilution and quantified by plate counting. A 200 μL aliquot of each dilution and a 200 μL heat-inactivated aliquot of each dilution were added to simulated fecal-contaminated water samples. For Kit method, following filtration, the membrane filter was saturated with 2 mL of 60 μM PMAxx solution (Biotium, USA) and incubated in darkness at 25 °C for 15 min. The filter was subsequently photoactivated using a 465 nm LED light array. For direct centrifugation, pelleted samples (180 μL) were treated with 20 μL of 600 μM PMAxx stock solution (final concentration: 60 μM), homogenized by vortex mixing (10 s), and dark-incubated (25 °C, 15 min). Photoactivation was performed using the PMA-Lite™ LED Photolysis Device for 5 min with continuous agitation. The samples were processed for nucleic acid extraction using the protocols described previously and extracted DNA from these samples was analyzed by ddPCR to detect and quantify *Shigella* strains. The performance of PMAxx under chlorination was assessed using simulated samples spiked with a serially diluted *S. flexneri* strain 301 suspension (2.05 × 10^8^ CFU/mL, plate-count verified). Samples were prepared with surface water, fecal suspension, and bacterial suspension, and divided into three groups: (1) all three components; (2) surface water and bacteria; and (3) surface water only. Each group was chlorinated for 30 min (≈5 mg/L Cl₂), then subjected to PMAxx treatment, DNA extraction, and ddPCR analysis. Biological replicates (*n* = 3) were treated as independent observational units. The Student’s *t*-test was performed to evaluate differences between experimental conditions, and *p* < 0.05 was considered statistically significant.

## Results

### Establishment ddPCR assay and optimization amplification condition of ddPCR

First, the common PCR was conducted using the primers designed in this study at 10 different anneal temperatures ranging from 51.0 °C to 59.7 °C. At all tested temperatures, a single band was consistently observed, indicating successful amplification. The cloning and sequencing analysis confirmed the presence of the target genes, including the *set*1A, *ss_methylase*, and *ipaBCD* gene, which further validated the effectiveness of the primers used in this study. Then, qPCR was performed at 60 °C using serially diluted plasmids ranging from 10^8^ to 10^0^ copies, which contained three target genes for analysis. Typical amplification curves were observed, confirming the effectiveness of the primers and probes (Supplemental Figure 2). Validation assays were performed using serial decimal dilutions (10^0^–10^8^) of plasmids with *set*1A, *ss_methylase* and *ipaBCD* genes at 60 °C with qPCR method (Supplemental Figure 2) with equimolar concentrations of with *ipaBCD*/*set*1A (*S. flexneri*) and *ipaBCD*/*ss_methylase* (*S. sonnei*) primer-probe sets. The *R*^2^ values of the amplification curves indicated good linearity and the two targets of the two sets showed almost consistent amplification efficiency (Supplemental Figure 2).

**Figure 1:**
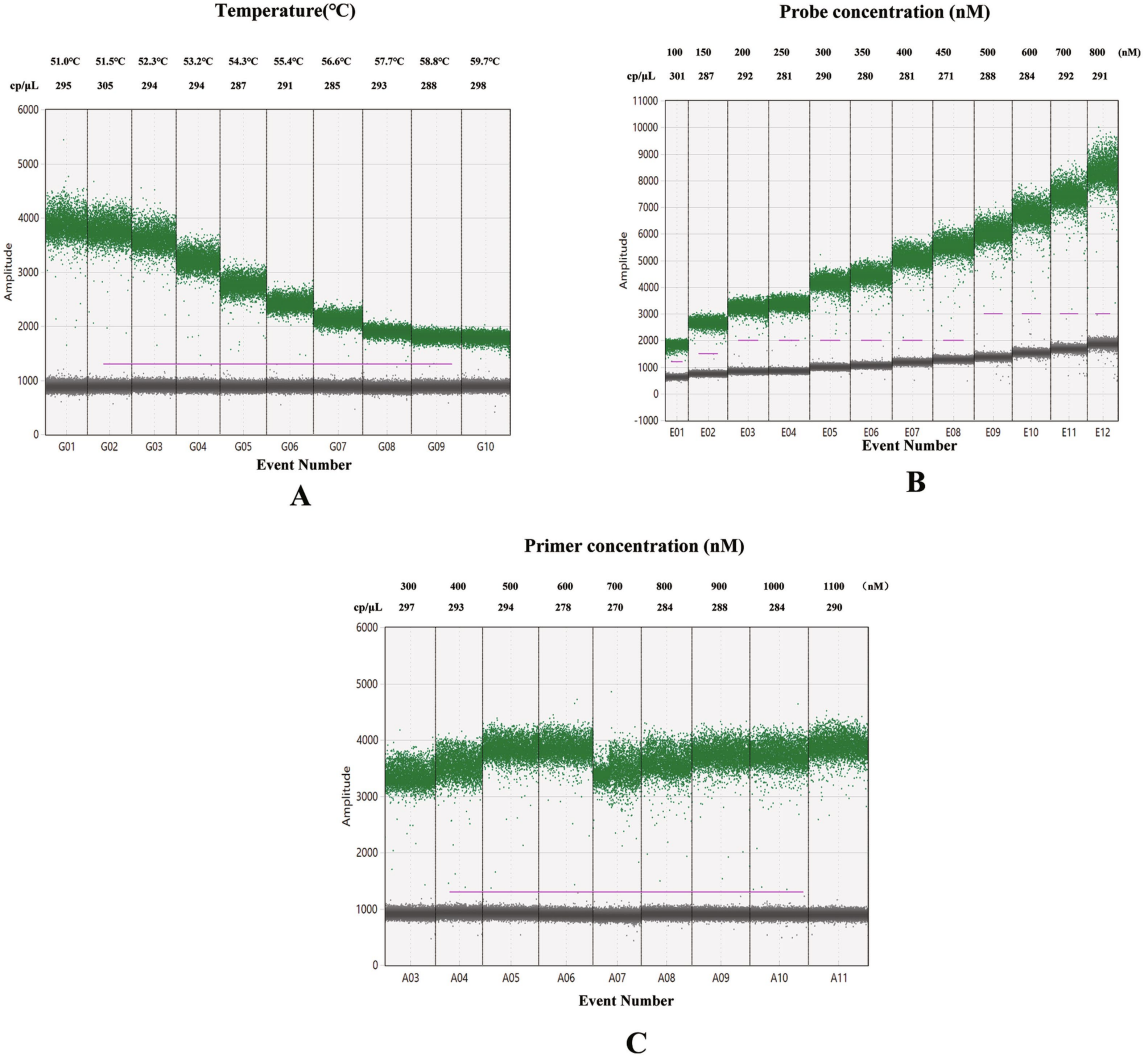
Scatter plots illustrated optimal amplification parameters for the *ipaBCD* gene in ddPCR assays: **(A)** Optimal annealing temperature of the *ipaBCD* gene in ddPCR; **(B)** Optimal primer concentrations of the *ipaBCD* gene in ddPCR; **(C)** Optimal probe concentrations of the *ipaBCD* gene in ddPCR. The horizontal axis represents the cumulative number of droplets, while the vertical axis indicates the fluorescence amplitude. Positive droplets are shown in blue and negative droplets in grey. The parameters of different reaction conditions and the number of positive droplets at various reaction conditions are labeled. cp/μL: Positive copies per μL.

To evaluate the amplification of the *ipaBCD* gene under various annealing temperatures, ddPCR was performed using conditions similar to those of standard PCR. Specifically, the probe concentration was set to 250 nM, primer concentration to 900 nM, and the DNA template was derived from *S. flexneri* strain 301, with a DNA concentration of 49.5 pg./μL. Positive droplets were obtained at all tested annealing temperatures, with clear separation between positive and negative droplets. As the annealing temperature increased, the number of positive copies increased, however, the fluorescence amplitude of the positive copies noticeably decreased as well (Figure 1A). Considering both fluorescence amplitude and the number of positive copies, an annealing temperature of 52.0 °C was chosen as optimal. The eight primer concentrations ranging from 300 to 1,000 nM with 100 nM interval were used to optimize the singleplex ddPCR assay of the *ipaBCD* gene by fixed probe concentration at 250 nM and annealing temperature at 52 °C (Figure 1B). Based on the desired amplification effectand the number of copies, the primers concentration of 500 nM was used (Figure 1B). Similarly, the probe concentration of 500 nM was determined as the optimal concentration (Figure 1C).

### The optimal reaction system of duplex ddPCR

**Figure 2:**
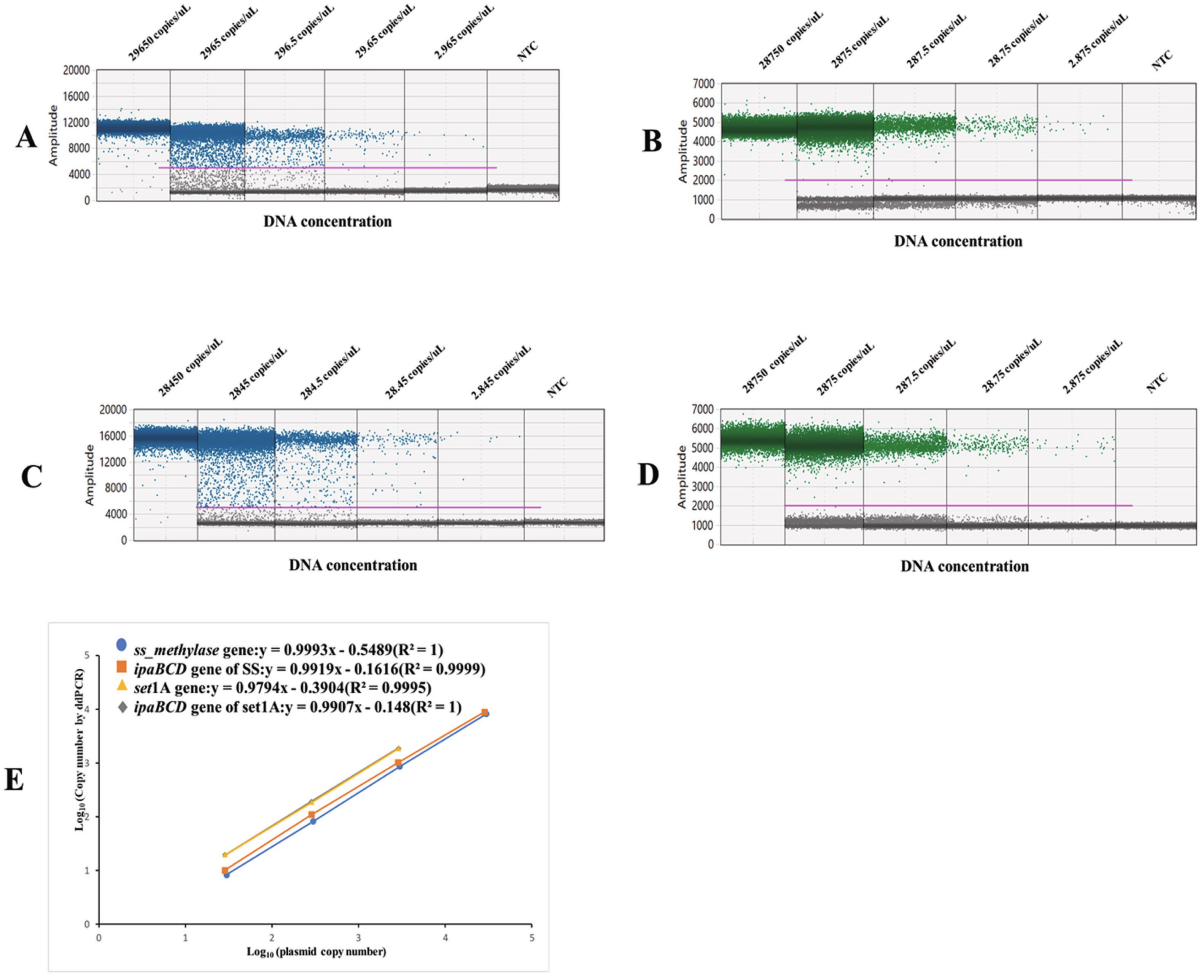
Performance of the ddPCR assays for detecting diluted recombinant plasmids of *Shigella*. **(A–D)** Amplification plots of the *set*1A, *ipaBCD* in the assay of *Shigella flexneri*, *ss_methylase* and *ipaBCD* in the assay of *Shigella sonnei* by ddPCR. Positive droplets are shown in blue and negative droplets in grey. **(E)** Linear quantification performance of duplex ddPCR assays for target genes. *X*-axis: Expected plasmid copy numbers Log^10^. *Y*-axis: Measured copy numbers Log^10^. **(F)** LoB and LoD values of the three gene targets. Each test was repeated in triplicate.

Based the results of qPCR, we initially assumed that the primer and probe concentrations could be kept consistent to ensure comparable performance in ddPCR assays. The primer and probe concentrations were adjusted based on the optimized conditions for the *ipaBCD* gene. The results after adding equal amounts of primers and probes are shown in Figure 2. As demonstrated, all three target genes (*ipaBCD*/*set*1A for *S. flexneri* and *ipaBCD*/*ss_methylase* for *S. sonnei*) were successfully amplified in their respective duplex ddPCR assays, exhibiting excellent and comparable amplification efficiency (Figures 2A–E). Notably, the amplification curves of *ipaBCD* were nearly identical between the two duplex systems (Figure 2E). The ddPCR assay achieved strong linearity (*R*^2^ = 0.99) across a four-log dynamic range for all targets (Figure 2E). Consequently, the optimized duplex system utilized equimolar concentrations (500 nM each) of primers and probes for all targets.

### Analytical sensitivity and specificity

In the qPCR assays, all three target genes were detectable at 10^0^ plasmid copies, with cycle threshold (Ct) values lower than those of no-template controls (NTCs) but differing by less than 1 Ct value compared to reactions containing 10^1^ plasmid copies (Supplemental Figure 2). Given the linear detection range constraints of qPCR methodology, the positive threshold was consequently established at 10^1^ plasmid copies.

The limits of blank (LoB) were determined through analysis of 35 negative controls (ddH₂O), yielding values of 0.29, 0.22, and 0.54 copies/reaction for *set*1A, *ss_methylase*, and *ipaBCD* targets, respectively (Supplemental Table 2). Sensitivity assessment employed triplicate testing of plasmid standards serially diluted from 10^5^ to 10^0^ copies/reaction (Figure 2). As demonstrated in Figure 2F, consistent three detection was achieved at 10^0^ copies/reaction across all replicates, with measured values exceeding the established LoB thresholds. Consequently, the threshold for positive detection for ddPCR was validated at 10^0^ copies/reaction.

To evaluate the specificity of the ddPCR assays, genomic DNA from 82 pathogenic bacterial strains (Supplemental Table 1) was used as templates. The results showed that *S. flexneri* and *S. sonnei* strains produced the expected positive droplets. Specifically, strain 042 was positive for the *set*1A gene but negative for *ipaBCD*, whereas enteroinvasive *E. coli* CMCC44825 was negative for *set*1A but positive for *ipaBCD* gene. All other pathogenic bacteria and the negative control generated only negative droplets.

### Optimization of PMAxx concentration and light-exposed time

The optimal PMAxx treatment conditions were established using *S. flexneri* strain 301 suspensions with three replicates. Eight PMAxx concentrations (0–120 μM) and six light exposure durations (0–30 min, 5-min intervals) were evaluated through differential qPCR analysis of viable and heat-inactivated cells (Supplemental Figure 3). For viable cells, Ct values remained stable (ΔCt < 1) at PMAxx concentrations ≤80 μM, with significant amplification inhibition observed at 100 μM (ΔCt = +2.27 vs. untreated control; *p* < 0.05, Student’s *t*-test). Non-viable cells exhibited dose-dependent increases in Ct values during qPCR analysis. Amplification inhibition was observed at the concentration of 10 μM PMAxx (ΔCt = +3.2 ± 0.4 vs. untreated controls; *p* < 0.05), with significant suppression achieved at 60 μM (*p* < 0.05). Consequently, 60 μM was selected as the optimal concentration, balancing effective dead-cell DNA suppression with minimal impact on viable cell detection (Supplemental Figure 3A). Light exposure optimization demonstrated equivalent inhibition efficacy between 5-min (*p* < 0.05 vs. untreated) and longer durations (*p* > 0.05, one-way ANOVA), leading to the selection of 5-min exposure (Supplemental Figure 3B).

### Comparison and optimization of DNA extraction methods from simulative water samples

A serially diluted *S. flexneri* strain 301, quantified as 2.10 × 10^8^ CFU/mL by plate counting method, was used to compare the three sample preparation methods: the Kit method, PEG precipitation, and Direct centrifugation. As evidenced by Table 2, the PEG precipitation method exhibited significantly reduced sensitivity (>10-fold lower) compared to alternative concentration techniques in simulated fecal-contaminated water samples. Furthermore, quantification results across dilution gradients demonstrated non-linear dynamics, indicating limited reliability for absolute quantification. Both the Kit method and Direct centrifugation were able to detect down to 10^0^ CFU of *S. flexneri*, but the copies number obtained with the Kit method was higher than that with Direct centrifugation at 10^2^ and 10^1^ level at both *set*1A and *ipaBCD* genes (Table 2). Therefore, in subsequent experiments, we focused only on the comparison between the Kit method and Direct centrifugation.

**Table 2:** Comparison of digital PCR performance using different nucleic acid extraction methods for Shigella detection in fecal-simulated water matrices.

Expected copies/Reaction (cp/μL)	Kit method		Direct centrifugation		*p* value*		PEG precipitation	
	*set*1A	*ipaBCD*	*set*1A	*ipaBCD*	*set*1A	*ipaBCD*	*set*1A	*ipaBCD*
2100	3,390.00	4,615.33	984.67	1,867.00	<0.05	0.0582	44.63	54.07
210	262.67	452.33	119.00	277.00	<0.0001	<0.0001	12.10	14.20
21	24.30	41.27	12.27	25.90	<0.05	<0.0009	0.71	0.94
2.1	2.78	4.49	6.21	8.24			0.96	1.18
Blank	0	0	0	0			0.11	0

^*^Compared with Kit method and direct centrifugation, Student’s *t*-tests.

### The application of PMAxx-ddPCR assay in simulated water samples

For simulated sample validation, 10-fold diluted viable cultures and equivalent concentrations of heat-inactivated *S. flexneri* (2.75 × 10^8^ CFU/mL by plate count) and *S. sonnei* (1.95 × 10^8^ CFU/mL) were processed. DNA extraction and analysis followed the established protocol, with quantitative results presented in Table 3. As demonstrated in Table 3, the inclusion of heat-killed bacteria did not compromise detection accuracy. Both the kit-based and direct centrifugation methods successfully discriminated viable *Shigella* cells, confirming PMAxx’s effective suppression of dead-cell DNA amplification. The kit method achieved single-digit sensitivity (<10 CFU/reaction), surpassing conventional qPCR by an order of magnitude. The direct centrifugation method demonstrated reduced sensitivity compared to the kit-based approach, with a detection limit of 10 viable bacterial cells per reaction. Notably, quantification results obtained by this method exhibited non-linear distribution characteristics. The ddPCR results demonstrated comparable mean copy numbers between samples with and without fecal matrix (i.e., surface water, feces, and bacteria vs. surface water and bacteria). Student’s t-test indicated no statistically significant difference between the two groups (*p* > 0.05), suggesting that the presence of fecal contamination did not compromise PMAxx treatment efficacy under the chlorination conditions tested (Supplemental Table 3).

**Table 3:** Performance of PMAxx-ddPCR method for viable *Shigella* detection in fecal-spiked water samples.

(A) The results of PMAxx-ddPCR assay for *Shigella flexneri*												
Expected copies/Reaction (cp/μL)	Kit method						Direct centrifugation					
	*set*1A			*ipaBCD*			*set*1A			*ipaBCD*		
	Mean	SD	CV	Mean	SD	CV	Mean	SD	CV	Mean	SD	CV
	(cp/r)	(cp/r)	(%)	(cp/r)	(cp/r)	(%)	(cp/r)	(cp/r)	(%)	(cp/r)	(cp/r)	(%)
2750.00	1,108.38	28.43	2.57	1,394.46	52.37	3.76	478.38	34.49	7.21	677.46	29.14	4.30
275.00	109.38	1.53	1.40	170.79	2.89	1.69	25.81	1.65	6.38	30.56	3.73	12.21
27.50	16.38	2.99	18.28	18.83	3.75	19.92	18.41	2.23	12.13	4.91	0.88	17.84
2.75	0.87	0.03	3.50	0.40	0.11	28.87	1.32	0.62	47.00	0.13	0.22	173.21

(B) The results of PMAxx-ddPCR assay for *S. sonnei*												
Expected copies/Reaction (cp/μL)	Kit method						Direct centrifugation					
	*ss_methylase*			*ipaBCD*			*ss_methylase*			*ipaBCD*		
	Mean	SD	CV	Mean	SD	CV	Mean	SD	CV	Mean	SD	CV
	(cp/r)	(cp/r)	(%)	(cp/r)	(cp/r)	(%)	(cp/r)	(cp/r)	(%)	(cp/r)	(cp/r)	(%)
1950.00	913.11	69.00	7.56	2,597.46	225.71	8.69	149.45	10.97	7.34	502.46	5.57	1.11
195.00	160.45	9.81	6.12	466.46	29.61	6.35	8.12	0.86	10.55	26.89	2.62	9.73
19.50	11.71	0.85	7.26	33.49	0.49	1.47	1.05	0.32	30.74	4.20	0.41	9.69
1.95	1.51	0.24	15.92	3.35	0.36	10.63	0.10	0.17	173.21	0.29	0.28	96.44

## Discussion

In this study, we developed and optimized two duplex PMAxx-ddPCR assays for simultaneous detection of viable *Shigella* strains in water. The developed assay simultaneously targets plasmid-borne and chromosomal markers to distinguish viable, pathogenic *Shigella* from non-viable cells or commensal bacteria. For both species, we selected *ipaBCD*—a single-copy virulence gene located on the large plasmid—which is widely recognized as a reliable detection target for waterborne *Shigella* (Faruque et al., 2002). The big plasmid is essential for pathogenicity, as only live *Shigella* retaining this plasmid can invade human intestinal epithelium. For species differentiation, *S. flexneri* detection incorporates *set*1A, a single-copy chromosomal gene originally characterized as encoding the ShET1 enterotoxin of *S. flexneri* (Henderson et al., 1999). The *set*1A gene was initially characterized as a serotype-specific marker for *S. flexneri* 2a strains. Subsequent epidemiological investigations have demonstrated a broader distribution of *set*1A across multiple *S. flexneri* serotypes (including 1b, 2b, and others), particularly among clinical isolates from diarrheal cases (Vargas et al., 1999; Yavzori et al., 2002). This expanded prevalence, especially in Chinese clinical populations, underscores its value as both a species-specific genetic marker and an indicator of clinical relevance for pathogenic *S. flexneri* detection, demonstrating its value as a species-specific marker with high clinical relevance (Fan et al., 2017). Similarly, *S. sonnei* detection employs *ss_methylase*, a conserved single-copy chromosomal gene (Shepherd James et al., 2000; Jiang et al., 2005). Specificity testing confirmed that this plasmid-chromosome dual-target strategy enables specific detection *Shigella flexneri* and *S. sonnei* strains.

The use of quantified plasmid standards—rather than genomic DNA extracted from bacterial strains—for optimizing duplex ddPCR assays was strategically employed to precisely evaluate primer-probe amplification efficiency. This approach circumvents potential inaccuracies arising from natural plasmid loss in *Shigella* cultures, where spontaneous loss of the large plasmid virulence existed during standard laboratory passage (Haidar-Ahmad et al., 2023). Systematic optimization of annealing temperature, primer-probe stoichiometry, and thermal cycling conditions (with particular attention to minimizing the “tailing” of positive droplets) enabled the method to achieve a positive detection of 1 plasmid copy per reaction. This represents a 10-fold improvement over qPCR using identical primers and probes. This sensitivity threshold was validated across three orders of magnitude (10^2^–10^0^ copies/μL), demonstrating linear quantification (*R*^2^ > 0.998) without cross-target interference (Figure 2).

The quantification of viable *Shigella* in water matrices faces two fundamental constraints: inherently low pathogen concentrations, and potent PCR inhibition by co-existing impurities. Our comparative evaluation of three nucleic acid extraction methods—kit method (DNeasy PowerWater kit), PEG precipitation, and direct centrifugation—revealed critical performance differentials. While PEG precipitation, widely adopted for viral pathogens (e.g., SARS-CoV-2 in wastewater), offers theoretical advantages for simultaneous bacterium-virus co-extraction, its recovery efficiency for *Shigella* proved inferior by one order of magnitude versus alternative methods (Table 2). The kit-based approach demonstrated optimal sensitivity but incurred substantial operational complexity and cost. Crucially, its multi-step workflow impedes seamless integration with viability staining—a limitation circumvented by direct centrifugation. Although centrifugation yielded lower DNA purity than kit extraction, ddPCR’s superior tolerance to inhibitory substances enabled equivalent quantification accuracy at clinically relevant concentrations (10^2^–10^5^ CFU/L). This operational simplicity, combined with compatibility with PMAxx treatment, positions centrifugation as a pragmatic solution for resource-limited settings. It is worth noting that the non-linear quantification observed with the direct centrifugation method may be attributed to variable inhibitor carryover, inconsistent recovery efficiency, or droplet partitioning artifacts. Method validation using spiked fecal-water samples confirmed reliable detection of viable plasmid-bearing strains at 10^0^ CFU/mL, establishing a robust framework for water safety monitoring. The observed quantitative differences between Tables 2, 3 and Supplemental Table 3 are likely attributable to PMAxx-mediated inhibition of dead-cell DNA amplification, thereby reducing enumeration errors in plate counts through selective elimination of non-viable bacterial signals.

There are still some limitations in our study. Firstly, although PMAxx treatment is widely used technique for selectively detecting cells with intact membranes, it serves as an indicator of membrane integrity rather than a direct measure of cultivability or infectivity. The method in this study could potentially underestimate the presence of non-culturable yet potentially infectious bacterial populations, a point important for public health risk assessment. Further consideration involves the specificity of our molecular targets. While the *set*1A gene is a prevalent marker, it is not universal across all *S. flexneri* serotypes, creating a potential for false negatives. Conversely, the *ipaBCD* virulence gene is not exclusive to *Shigella* but is also carried by enteroinvasive *E. coli* (EIEC). Consequently, we recommend that environmental samples testing positive for *ipaBCD* but negative for *set*1A or *ss_methylase* undergo confirmatory sequencing for definitive pathogen identification. Additionally, the interpretation of quantitative results is constrained by the variable copy number of the virulence plasmid within and across bacterial cells, as plasmid copy number can fluctuate and plasmid loss may occur.

In conclusion, this study advances the application of PMA-ddPCR for monitoring waterborne *Shigella* by introducing a key methodological integration. We developed a duplex assay that concurrently evaluates bacterial membrane integrity and pathogenic potential through parallel detection of chromosomal and plasmid-borne virulence genes. Combined with a systematically optimized, field-adaptable sample processing workflow, this approach provides a refined tool not only for improving public health risk assessment in water surveillance but also for future studies on the environmental persistence and adaptability of these pathogens.

## Data Availability

The original contributions presented in the study are included in the article/Supplementary material, further inquiries can be directed to the corresponding author.

## Footnotes

1
https://blast.ncbi.nlm.nih.gov/Blast.cgi
