# Supplementary material for: Development of PMAxx-droplet digital PCR method for the absolute quantification of viable Shigella flexneri and Shigella sonnei strains in water
Source: Front Microbiol. 2026 Mar 6;17:1769049. doi: 10.3389/fmicb.2026.1769049 (PMC13002800; doi:10.3389/fmicb.2026.1769049)
Supplement: Supplementary file 1 [file Data_Sheet_1.PDF]

Supplemental Figure 1. The workflow for processing simulated fecal-contaminated water samples.

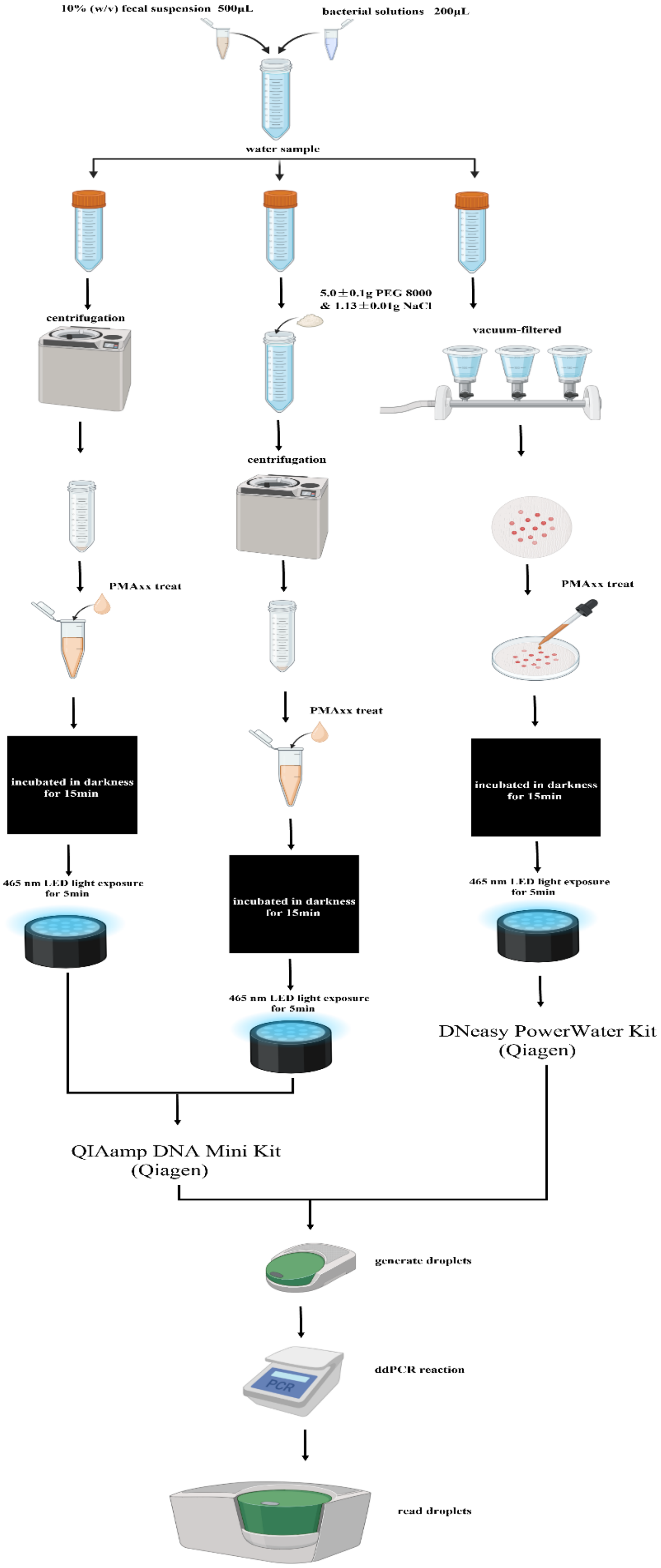

Supplemental Figure2. qPCR standard curves for plasmid standards containing *Shigella flexneri* and *S. sonnei* target genes.

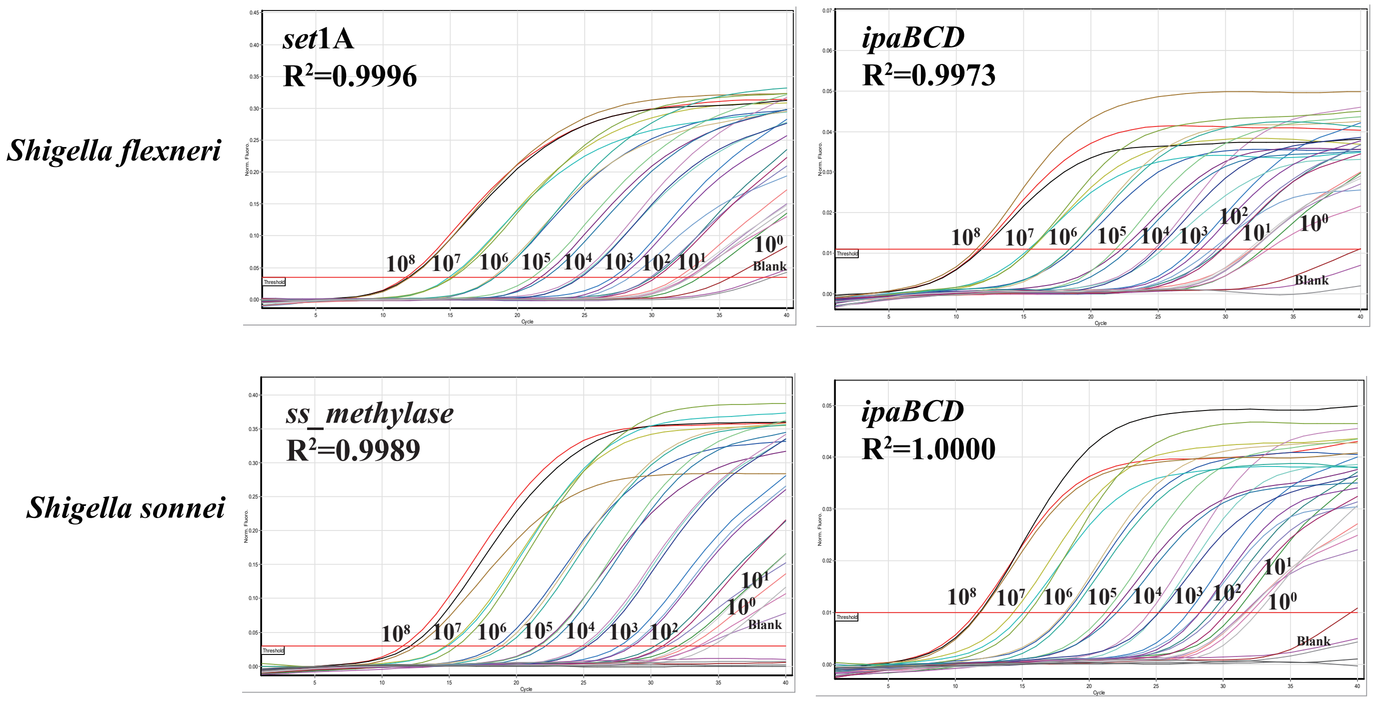

Supplemental Figure 3. Optimization of PMAxx treatment conditions. Error bars represent standard deviations from triplicate measurements.
(A) Effect of PMAxx concentration (10-120 μM) on viable (*S. flexneri* 301) versus heat-inactivated cell suspensions.
(B) Determination of optimal photoactivation duration for PMAxx-treated heat-killed cells. Untreated controls (Blank) were included for reference.

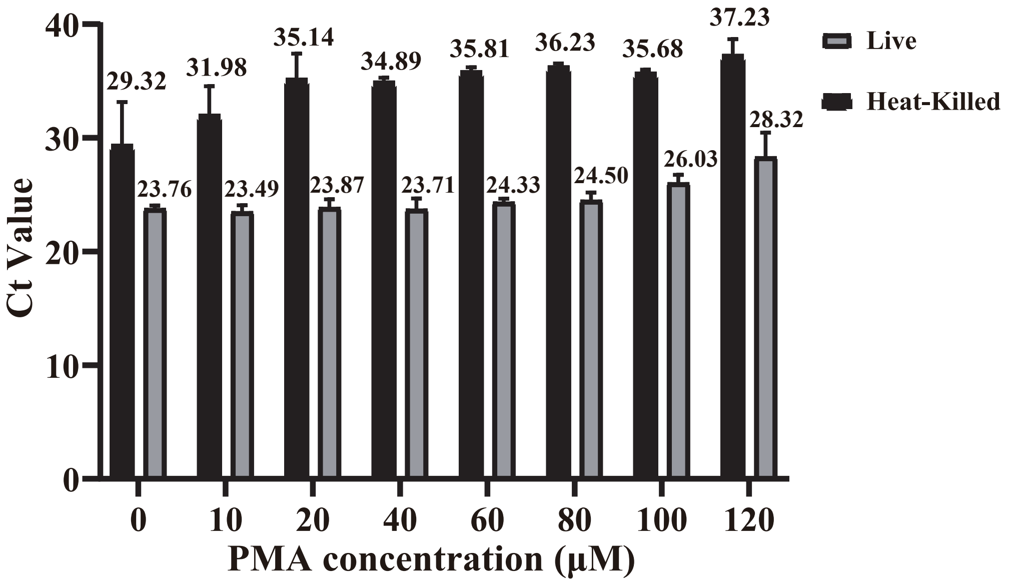

A

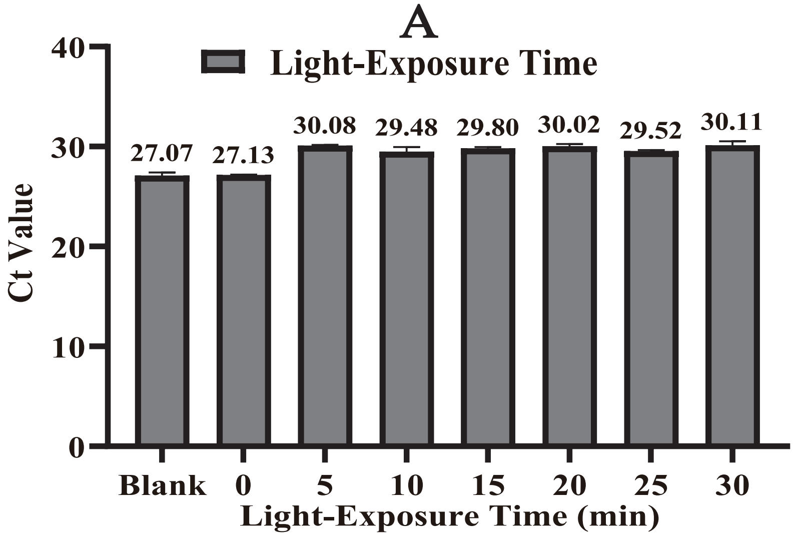

B

Supplemental Table 1. Strains used in this study and the results of ddPCR.

Bacteria	Strain no./source[a]	No. of strains	ddPCR results[d]		
			*set*1A	*ss_methylase*	*ipaBCD*
Shigella sonnei phase I	CMCC 51081	1	−	+	+
S. sonnei phase I	Isolated strain	20	−	+	+
S. sonnei phase II	CMCC M6381	1	−	+	−
S. sonnei phase II	Isolated strain	10	−	+	−
S. flexneri	301	1	+	−	+
S. flexneri	Isolated strain	20	+	−	+
enteroinvasive *E. coli*	CMCC 44825	1	−	−	+
enterohemorrhagic *E. coli* O157:H7	ATCC 700927	1	−	−	−
enteroaggregative *E. coli*	042	1	−	−	−
enteropathogenic *E. coli*	2348/69	1	−	−	−
enterotoxigenic *E. coli*	10407	1	−	−	−
uropathogenic *E. coli*	UPEC 536	1	−	−	−
Plesiomonas shigelloides	CHPC 1.5797	1	−	−	−
Listeria monocytogenes	ATCC BAA-679	1	−	−	−
Salmonella enterica serovar Paratyphi A	CMCC 50001	1	−	−	−
S. enterica serovar Choleraesuis	ATCC BAA-664	1	−	−	−
S. typhimurium	CMCC 50013	1	−	−	−
Enterobacter cloacae	ATCC 700323	1	−	−	−
Vibrio parahemolyticus	ATCC 17802	1	−	−	−
Yersinia enterocolitica	Isolated strain	1	−	−	−
Citrobacter freundii	ATCC 8090	1	−	−	−
Pseudomonas aeruginosa	ATCC 27853	1	−	−	−
Acinetobacter baumannii	ATCC 17978	1	−	−	−
Enterococcus faecium	CGMCC1.2135	1	−	−	−
E. faecalis	CGMCC1.2136	1	−	−	−
E. durans	CGMCC 1.2284	1	−	−	−
E. avium	CGMCC 1.2505	1	−	−	−
E. hirae	CGMCC 1.2140	1	−	−	−
E. mundtii	CGMCC 1.2486	1	−	−	−
Staphylococcus aureus subsp. aureus	ATCC 25923	1	−	−	−
Streptococcus pneumoniae	ATCC 49619	1	−	−	−
S. alactolyticus	ATCC 43077	1	−	−	−
S. pyogenes	ATCC 19615	1	−	−	−
Stenotrophomonas maltophilia	CGMCC 1.1788	1	−	−	−
Burkholderia cepacia	CGMCC 1.1813	1	−	−	−

a, CMCC, National center for Medical Culture Collections, ATCC, American Type Culture Collection, CGMCC, China General Microbiological Culture Collection Center, CPHC, National Pathogen Resource

b, +, positive; −, negative

Supplemental Table 2. Detection Results of Blank Samples (ddH_2O) and LoB Calculation of ddPCR.

Number	Blank results (copies/μL)		
	ipaBCD	*ss_methylase*	*set*1A
N1	0	0	0
N2	0	0	0
N3	0	0	0
N4	0	0	0
N5	0	0	0
N6	0	0	0
N7	0	0	0
N8	0	0	0
N9	0	0	0
N10	0	0	0
N11	0	0	0
N12	0	0	0
N13	0	0	0
N14	0	0	0
N15	0	0	0
N16	0	0	0
N17	0	0	0
N18	0	0	0
N19	0	0	0
N20	0	0	0
N21	0.07	0	0
N22	0.07	0	0
N23	0.09	0	0
N24	0.09	0	0
N25	0.11	0	0
N26	0.11	0	0
N27	0.11	0	0
N28	0.13	0.05	0
N29	0.15	0.08	0
N30	0.15	0.11	0.11
N31	0.22	0.12	0.11
N32	0.24	0.12	0.12
N33	0.29	0.14	0.22
N34	0.43	0.25	0.26
N35	0.48	0.35	0.40
LoB[a]	0.395[b]	0.22[c]	0.25[d]

Shapiro-Wilk test revealed that the data for all three target genes significantly deviated from normality ($P < 0.05$).

[a]As per the CLSI EP17-A2 guideline, Rank Position=0.5+35• 0.95=33.75

[b]LoB (*ipaBCD* gene) = N33 + 0.75• (N34 - N33) = 0.43+0.75• (0.43-0.29) = 0.395 copies/μL

[c]LoB (*ss_methylase* gene) = N33 + 0.75• (N34 - N33) = 0.14+0.75• (0.25-0.14) = 0.22copies/μL

[d]LoB (*set*1A gene) = N33 + 0.75• (N34 - N33) = 0.26+0.75• (0.26-0.22) = 0.25 copies/μL

Supplemental Table 3. ddPCR quantification of *S. flexneri* strain in simulated water samples after chlorination and PMAxx treatment.

	*Set*1A				*ipaBCD*			
	Mean (cp/r)	SD (cp/r)	CV (%)		Mean (cp/r)	SD (cp/r)	CV (%)	
surface water, fecal suspension and bacteria	132.67	30.55	23.03	$P > 0.05$	305.00	75.72	24.83	$P > 0.05$
surface water and bacteria	148.67	17.90	12.04		255.33	53.30	20.88	
surface water	0.22	0.32	146.21		0.44	0.32	71.61	
